# Towards Understanding the Lymph Node Response to Skin Infection with Saprophytic *Staphylococcus epidermidis*

**DOI:** 10.3390/biomedicines10051021

**Published:** 2022-04-28

**Authors:** Marta Cąkała-Jakimowicz, Monika Puzianowska-Kuznicka

**Affiliations:** 1 Department of Human Epigenetics, Mossakowski Medical Research Institute, 02-106 Warsaw, Poland; 2 Department of Geriatrics and Gerontology, Medical Centre of Postgraduate Education, 01-813 Warsaw, Poland

**Keywords:** *Staphylococcus epidermidis*, infection, lymph node, innate immunity, adaptive immunity, immune cells

## Abstract

In individuals with lymphedema, diabetic foot, or other diseases, infections with saprophytes are common. The response of major cell subpopulations in the draining lymph nodes to skin infection with *Staphylococcus epidermidis* was assessed using the rat model. After massive subepidermal infection, a cytometric evaluation showed an increase in cytotoxic and helper T lymphocytes and major subpopulations of the innate immune response. Three weeks later, signs of inflammation reduction with an increase in the content of memory T helper lymphocytes and effector memory T cytotoxic lymphocytes were observed. After skin re-infection, a rapid response of cytotoxic, helper, and memory T lymphocytes, memory B lymphocytes and plasmablasts, and macrophages was detected. In addition, a reduction in the number of naïve B lymphocytes, activated MHC class II+ cells, and some cells of the innate immune system was observed. T regulatory lymphocyte response after the initial and secondary *S. epidermidis* skin infection was not detected. The morphometric evaluation showed significant changes in the main cell subpopulations in each functional zone of the node and then confirmed the efficient elimination of the administered antigen, as evidenced by the observations on day 28. Notably, after re-infection, the cellular response did not exceed the level after the initial infection and was reduced in many cell subpopulations. Understanding how the lymph nodes eliminate *S. epidermidis* can provide valuable insights into creating immunological therapies against infections with saprophytes.

## 1. Introduction

Skin is permanently in contact with an environment rich in microorganisms and chemicals. The skin is colonized by saprophytic bacterial flora, including *cocci*, which constitute the majority of all bacterial strains isolated [1]. Among them, *Staphylococcus epidermidis* and other coagulase-negative strains were identified in most cases. In a previous study, *S. epidermidis* was grown from the lymph cultures of lower limbs in 12% of healthy volunteers [2]. By the production of antimicrobial proteins (AMP) and the stimulation of keratinocytes to produce these proteins, *S. epidermidis* plays a significant role in the control of pathogenic bacteria and viruses [3,4,5,6]. It also promotes wound healing [5]. Other strains found in minor amounts on the skin are *Corynebacterium*, *Propionibacterium*, *Brevibacterium*, and *Micrococcus* [7,8]. Tolerance to saprophytic bacteria inhabiting the skin develops during the neonatal period [9]. At that time, the commensals are recognized by the immune cells even without skin damage. The reaction is systemic, as evidenced by the substantial expansion of regulatory CD4+ T lymphocytes (Treg) specific for *S. epidermidis* used in animal model studies. This, subsequently, prevents commensal-specific effector cell responses, neutrophil infiltration into the skin, and reduces inflammation. In mice, the Treg activation is significantly more robust in neonates than in adult animals [10]. After the first contact with commensal microbes (e.g., due to microtrauma), immunological memory is probably created, allowing for quick removal (inactivation) of the re-penetrating antigen. Although microbial skin penetration is a continuous process, lymph nodes eliminate the drained microorganisms, and the small amounts of saprophytic bacteria do not elicit an inflammatory response [10,11,12,13]. When a large mass of bacteria penetrates the skin and subcutaneous tissue, the draining lymph nodes increase in size. If the inflammatory process lasts for an extended time (e.g., in a non-healing wound), apoptosis and necrosis of the lymph nodes occur, followed by their fibrosis [14,15,16]. The lymphatic vessels also become fibrotic. Lymphatic stasis develops, leading to local edema [16,17]. Lack of lymphatic drainage allows saprophytic bacteria to multiply in the tissue and favors the conversion of their phenotype into pathogenic, possibly due to environmental changes [18,19,20,21]. *S. epidermidis* is one of the microorganisms most frequently identified in cultures of tissue fluid, lymph, inguinal lymph nodes, skin, and subcutaneous tissue of patients with lymph stasis and acute form of dermato-lymphangio-adenitis [2,17,19,20,21]. Similarly, methicillin-resistant *S. epidermidis* has been identified in patients with genital lymphangiectasia and recurrent cellulitis [22]. In addition, an examination of bacterial isolates from atopic dermatitis patients revealed that *S. epidermidis* is present in the most severely affected skin regions [23]. Notably, *S. epidermidis* can induce the production of pro-inflammatory cytokines (e.g., TNFα, IL-6 and IL-8, IL-1β, and IL-12) in vitro [24].

These and other clinical observations indicate a crucial role of the skin-draining lymph nodes in recognizing and eliminating skin-derived bacteria. Therefore, knowing the details of the lymph node response to saprophytes under physiological and pathological conditions is essential. In our previous study, *S. epidermidis* induced the most potent immune changes in rat skin, afferent lymphatic vessels, and popliteal lymph nodes among seven saprophytic bacterial strains isolated from healthy human skin [25]. Therefore, in the current study, the detailed response of the key lymph node zones to the massive and secondary infections with *S. epidermidis* was investigated, and the phenotypes of memory cells involved in *S. epidermidis* elimination were examined.

## 2. Materials and Methods

### 2.1. Animals

The experiments were performed on 40 adult male Wistar–WAG rats weighing 230–250 g from which 80 popliteal lymph nodes were isolated. The experiments were performed under general anesthesia with a 3.6% chloral hydrate administered intraperitoneally at a dose of 1 mL per 100 g of body weight.

### 2.2. Bacterial Infection

**Figure 1:**
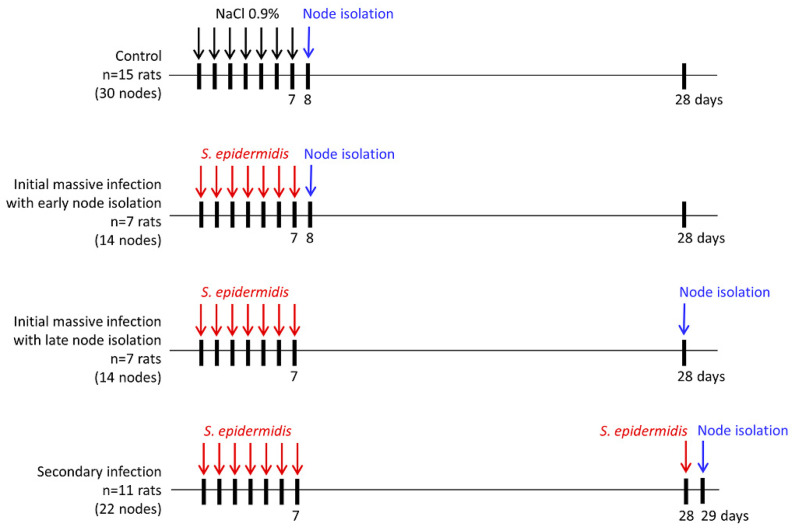
Experimental groups.

Coagulase-negative *S. epidermidis* strain resistant to penicillin but sensitive to other standard antibiotics isolated from healthy skin of human lower limbs was used in the present study. *S. epidermidis* bacteria were grown on Mueller-Hinton Agar (Oxoid Limited, Basingstoke, U.K.), and 6 × 10^7^ cells suspended in 0.1 mL of 0.9% NaCl were administered sub-epidermally in the dorsum of rat hind paws. In control animals, 0.1 mL of 0.9% NaCl was injected. Popliteal lymph nodes were harvested and weighed, and the number and types of cells were evaluated. The time-course of the experiment was designed based on previously published data on the dynamics of changes in the innate and acquired immune responses after primary and secondary antigen exposure [26,27]. The schematic presentation of experimental groups is shown in Figure 1.

### 2.3. Cells Isolation

Freshly isolated popliteal lymph nodes were weighed on a laboratory scale (Mettler Toledo, XS205 Dual Range, Warsaw, Poland). Free-floating immune cells not attached to the node’s stroma or capsule were isolated by gentle tissue fragmentation, pipetting, and washing in RPMI 1640 (Thermo Fisher Scientific, Waltham, MA, USA) supplemented with 5% fetal calf serum (FCS). The suspension was filtered through an intravenous in-line filter (Farmachin, Sofia, Bulgaria) to remove tissue debris and washed two times in RPMI 1640 with 5% FCS. Cells were counted in a Bürker chamber. Cell viability was assessed using trypan blue.

### 2.4. Flow Cytometry

Changes in cell subpopulations were evaluated using standard procedures with the LSR flow cytometer (Becton Dickinson, San Jose, CA, USA) and the Cell Quest Pro program (Becton Dickinson). Phenotypes of the popliteal lymph node cells were established based on their positive staining for the presence of a given antigen (Table 1, Supplementary Table S1).

### 2.5. Immunohistochemistry and Quantitative Analysis of Cell Phenotypes

Freshly isolated popliteal lymph nodes were frozen in an acetone/dry ice bath, sliced into 5 µm sections, and placed onto poly-α-lysine-coated slides. After drying, slides were fixed for 10 min in chilled acetone, blocked for 20 min with 50% goat serum in TBS, and stained for 50 min with anti-rat monoclonal antibodies: CD43 (W3/13) 1:100, CD4 (W3/25) 1:100, CD8 (OX8) 1:100, B 1:100, OX62 1:30, CD68 (ED1) 1:100, CD31 1:50, CD90 (OX7) 1:70, HiS48 1:100, MHC class II (OX6) 1:100, CD54 1:50 (Serotec, Ltd., Kidlington, U.K., Table 1, Supplementary Table S1) or with TBS (negative control). After washing, the stained cells were visualized using the LSAB 2 AP complex (Dako Denmark, Glostrup, Denmark) and alkaline phosphatase substrate. Finally, the sections were stained with hematoxylin. The slides were analyzed using a BX40 light microscope (Olympus, Hamburg, Germany) with the Microimage^TM^ version 3.0 software for Windows (Olympus). The total area of cells positively stained with a specific antibody was determined in the follicles in addition to the subcapsular, paracortical, and medullary zones, and presented as the percentage of the whole area of a given node zone observed in one field of view. Three fields of view per animal per node zone were evaluated at 200× magnification.

### 2.6. Statistical Analysis

Statistical analyses were performed using the SAS program [28]. For Microimage data, a preliminary analysis of the differences between test groups and types of infections was performed using a generalized mixed linear model in which the rat was a random factor (SAS/GLIMMIX procedure). To identify differences, the effects of infection were compared for each node zone separately using Tukey’s analysis of variance and multiple comparisons (SAS/GLIMMIX procedure), which were performed at significance levels α = 0.05, α = 0.01, and α = 0.001. In the flow cytometry experiments, the analyzed samples contained a mix of cells from several rats. Therefore, the weighted sum of squares (SAS/GLM procedure) was used to analyze variance. As described above, multiple comparisons between types of infection were made at three levels of significance. A *p*-value < 0.05 was considered statistically significant.

## 3. Results

### 3.1. The Lymph Node Mass and Cell Number after Skin Infection with S. epidermidis

**Figure 2:**
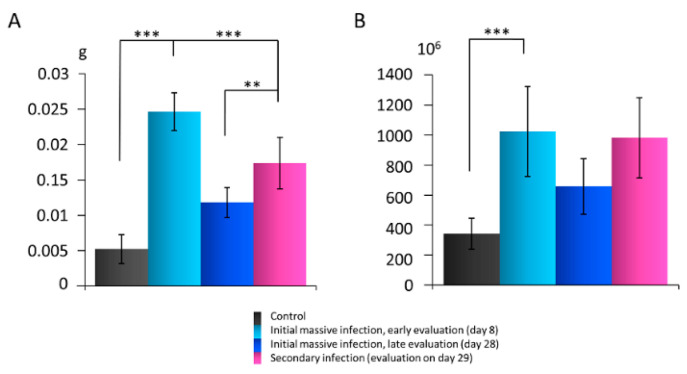
Rat popliteal lymph nodes after *S. epidermidis* skin infection (*n* = 32 rats, 64 nodes). (**A**) Changes in the lymph node mass (g). (**B**) Changes in the lymph node immune cell number. Results are presented as mean ± standard deviation (SD). ** *p* < 0.01; *** *p* < 0.001.

Evaluation on day 8, 24 h after the last administration of *S. epidermidis* during the initial massive skin infection cycle, showed the mass of the popliteal lymph nodes significantly increased compared with the controls administered 0.9% NaCl. However, the node mass decreased on day 28 (late isolation of the nodes). Secondary infection on day 28 resulted in a rapid, significant increase in the node weight, as evidenced 24 h after bacteria administration (day 29). Still, this increase was significantly less prominent than one day after the initial infection (Figure 2A).

After isolation, cell viability was 98%. Initial infection with *S. epidermidis* led to a significant increase in the total immune cell number per g of the lymph node mass on day 8. The cell number declined on day 28 and rapidly increased again one day after re-infection (day 29; Figure 2B).

### 3.2. Quantitative Changes in Cell Subpopulations in Lymph Node Zones after S. epidermidis Infection

**Figure 3:**
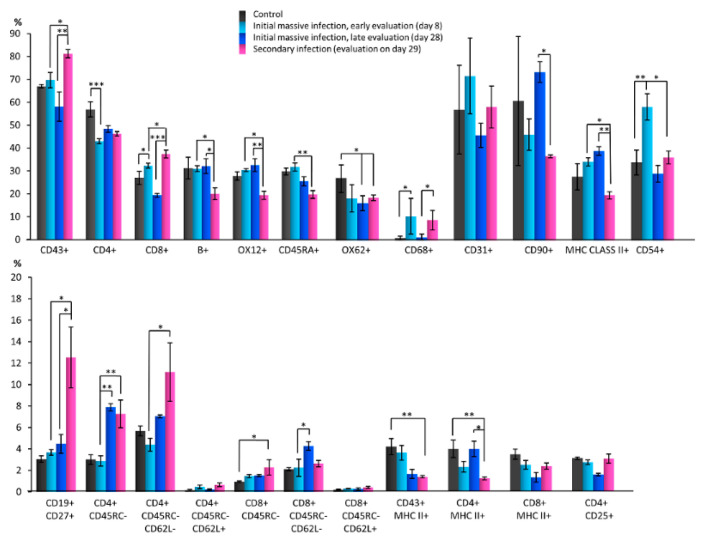
Changes in cell subpopulations of the popliteal lymph nodes in response to *S. epidermidis* skin infection assessed using a flow cytometry (*n* = 32 rats, 64 nodes). CD43+: T lymphocytes; CD4+: T helper lymphocytes, monocytes; CD8+: T cytotoxic lymphocytes; OX12+, CD45RA+, B+: B lymphocytes; OX62+: dendritic cells; CD68+: macrophages, monocytes; CD31+: PECAM-1, endothelial cells; CD90+: stem cells, thymocytes, immature B cells; MHC class II+: activated antigen-presenting cells; CD54+: dendritic, endothelial, ICAM-1+ cells; CD43+ MHC class II+, CD4+ MHC class II+, CD8+ MHC class II+: activated T lymphocytes; CD4+ CD25+: T regulatory lymphocytes; CD19+ CD27+: memory B cells, plasmablasts; CD4+ CD45RC- CD62L-/+: effector/central memory T helper lymphocytes; CD8+ CD45RC- CD62L-/+: effector/central memory T cytotoxic lymphocytes. The test results are presented as means ± standard deviation (SD). * *p* < 0.05; ** *p* < 0.01; *** *p* < 0.001.

First, *S. epidermidis* skin infection-related changes in cell subpopulations were evaluated in detail using flow cytometry. In each experiment, 10,000 cells were assessed and normalized to 100%. After the initial massive skin infection with *S. epidermidis*, a statistically significant increase in the percentage of T cytotoxic lymphocytes (CD8+), macrophages and monocytes (CD68+), dendritic, endothelial and ICAM-1+ cells (CD54+) was observed on day 8 of the experiment compared with the controls administered 0.9% NaCl solution. A significant reduction in the percentage of T helper lymphocytes (CD4+) was also detected. Furthermore, a non-significant increase in the percentage of B lymphocytes (OX12+), memory B cells and plasmablasts (CD19+ CD27+), central memory T helper lymphocytes (CD4+ CD45RC- CD62L+), and memory T cytotoxic lymphocytes (CD8+ CD45RC-) was observed, in addition to a non-significant reduction in the percentage of activated T helper lymphocytes (CD4+ MHC class II+), activated cytotoxic T lymphocytes (CD8+ MHC class II+), T regulatory lymphocytes (CD4+ CD25+), and effector memory T helper lymphocytes (CD4+ CD45RC- CD62L-) (Figure 3).

On day 28 after experiment initiation, statistically significant differences were not found in the percentage of the studied cell subpopulations compared with the controls obtaining 0.9% NaCl except for cells expressing OX62, whose population significantly decreased. However, a non-significant increase was observed in the proportion of B lymphocytes (OX12+), activated antigen-presenting cells (MHC class II+), plasmablasts and memory B cells (CD19+ CD27+), memory T helper lymphocytes (CD4+ CD45RC-), effector memory T helper lymphocytes (CD4+ CD45RC- CD62L-), memory T cytotoxic lymphocytes (CD8+ CD45RC-) and effector memory T cytotoxic lymphocytes (CD8+ CD45RC- CD62L-) compared with the controls. In addition, a non-significant reduction was demonstrated in the proportion of CD43+ T lymphocytes, T helper (CD4+) and T cytotoxic lymphocytes (CD8+), naïve B cells (CD45RA+), activated T lymphocytes (CD43+ MHC class II+), activated T cytotoxic lymphocytes (CD8+ MHC class II+) and T regulatory lymphocytes (CD4+ CD25+) (Figure 3).

Notably, a statistically significant increase was detected in the percentage of memory T helper lymphocytes (CD4+ CD45RC-) and effector memory T cytotoxic lymphocytes (CD8+ CD45RC- CD62L-) on day 28 compared with day 8. In addition, a non-significant increase was observed in the proportion of T helper lymphocytes (CD4+), stem cells, thymocytes and immature B cells (CD90+), activated antigen-presenting cells (MHC class II+), effector memory T helper lymphocytes (CD4+ CD45RC- CD62L-) and activated T helper lymphocytes (CD4+ MHC class II+), and a non-significant reduction in the percentage of T lymphocytes (CD43+), T cytotoxic lymphocytes (CD8+), naïve B cells (CD45RA+), macrophages and monocytes (CD68+), endothelial cells (CD31+), dendritic, endothelial, and ICAM-1+ cells (CD54+), activated T lymphocytes (CD43+ MHC class II+), activated T cytotoxic cells (CD8+ MHC class II+), T regulatory lymphocytes (CD4+ CD25+) and central memory T helper lymphocytes (CD4+ CD45RC- CD62L+) (Figure 3).

A statistically significant increase was observed in the percentage of CD43+ T lymphocytes, T cytotoxic lymphocytes (CD8+), plasmablasts and B memory cells (CD19+ CD27+), memory T helper lymphocytes (CD4+ CD45RC-), and effector memory T helper lymphocytes (CD4+ CD45RC- CD62L-), and statistically significant reductions in the percentage of B cells labeled with three different antibodies (B+ or OX12+ or CD45RA+), activated antigen-presenting cells (MHC class II+), dendritic, endothelial and ICAM-1+ cells (CD54+) 1 day after skin re-infection with *S. epidermidis* (day 29, Figure 1), compared with day 8 after initial infection. In addition, a non-significant reduction in the percentage of stem cells, thymocytes, and immature B cells (CD90+), activated T lymphocytes (CD43+ MHC class II+) and activated T helper lymphocytes (CD4+ MHC class II+), and a non-significant increase in the percentage of T helper lymphocytes (CD4+) were observed (Figure 3).

Notably, 24 h after *S. epidermidis* re-infection (day 29 vs. day 28), a statistically significant increase was observed in the percentage of CD43+ T lymphocytes, T cytotoxic lymphocytes (CD8+), macrophages and monocytes (CD68+), and plasmablasts and B memory cells (CD19+ CD27+). In addition, a statistically significant reduction was detected in the proportion of B cells (B+, OX12+), stem cells, thymocytes and immature B lymphocytes (CD90+), activated antigen-presenting cells (MHC class II+), and activated T helper cells (CD4+ MHC class II+). A non-significant increase was observed in the proportion of dendritic, endothelial and ICAM-1+ cells (CD54+), activated T cytotoxic lymphocytes (OX8+ MHC class II+), T regulatory lymphocytes (CD4+ CD25+), effector memory T helper lymphocytes (CD4+ CD45RC- CD62L-), central memory T helper lymphocytes (CD4+ CD45RC- CD62L+), and memory T cytotoxic lymphocytes (CD8+ CD45RC-), in addition to a non-significant reduction in the percentage of naïve B lymphocytes (CD45RA+) and effector memory T cytotoxic lymphocytes (CD8+ CD45RC- CD62L-) (Figure 3).

Next, individual lymph node zones were analyzed. Microscopic analysis using the Microimage^TM^ program revealed that after the initial massive skin infection with *S. epidermidis*, a significant increase in the percentage of surface area covered by CD43+ T lymphocytes, T helper lymphocytes (CD4+), T cytotoxic lymphocytes (CD8+), B lymphocytes, macrophages and monocytes (CD68+), granulocytes (HiS48+), stem cells, thymocytes and immature B cells (CD90+), activated antigen-presenting cells (MHC class II), and CD54+ dendritic, endothelial, and ICAM-1+ cells was observed in the subcapsular sinuses evaluated on day 8 compared with the nodes of animals administered 0.9% NaCl. In contrast, there was a reduction in the surface area covered by CD31+ endothelial cells. Notably, on day 28, the surface covered by cells expressing all tested antigens returned to its original size and was similar to control nodes. One day after secondary skin infection (day 29), the subcapsular sinus showed an increase in CD54-expressing dendritic, endothelial, and ICAM-1+ cells compared with days 8 and 28 after the initial infection. A decrease was also detected in the surface area covered by dendritic cells (OX62+), macrophages and monocytes (CD68+), granulocytes (HiS48+), and endothelial cells (CD31+) compared with the early observation (day 8) after the initial infection (Supplementary Figures S1–S4).

In the follicles, the area covered by B lymphocytes increased after the initial massive *S. epidermidis* skin infection as shown on experimental day 8 compared with the follicles in controls administered 0.9% NaCl; however, the significance level was not reached. The surface area covered by CD43+ T lymphocytes, T helper lymphocytes (CD4+), macrophages and monocytes (CD68+), endothelial cells (CD31+), stem cells, thymocytes and immature B cells (CD90+), granulocytes (HiS48+), activated antigen-presenting cells (MHC class II+), and CD54+ dendritic, endothelial, and ICAM-1+ cells also increased on day 8 compared with control nodes. However, on day 28, the surface covered by cells expressing all tested antigens became similar to that observed in control nodes. After secondary skin infection, a significant increase in the area positive for CD54-expressing dendritic, endothelial, and ICAM-1+ cells was observed compared with observations made on days 8 and 28 after the initial infection. In contrast, the area covered by endothelial cells (CD31+) was smaller compared with day 8 but larger than on day 28 after the initial infection (Supplementary Figures S5 and S6).

After the initial massive infection with *S. epidermidis*, the early evaluation of the paracortex showed a significant increase in the surface area stained for the presence of CD43+ T lymphocytes, T helper lymphocytes (CD4+), T cytotoxic lymphocytes (CD8+), B lymphocytes, dendritic cells (OX62+), macrophages and monocytes (CD68+), granulocytes (HiS48+), stem cells, thymocytes and immature B lymphocytes (CD90+), MHC class II+ cells, and CD54-positive dendritic, endothelial, and ICAM-1+ cells compared with controls administered 0.9% NaCl, but the significant changes were no longer present on day 28. One day after secondary skin infection, the paracortical zone showed a decrease in the surface area positively stained for the presence of T helper cells (CD4+), dendritic cells (OX62+), stem cells, immature B cells and thymocytes (CD90+), and endothelial cells (CD31+) compared with the early evaluation (day 8) after the initial infection. In contrast, an increase in the area covered by T cytotoxic lymphocytes (CD8+), dendritic cells (OX62+), granulocytes (HiS48+), and activated antigen-presenting cells (MHC class II+) was observed compared with day 28 after the initial infection (Supplementary Figures S7–S10).

**Figure 4:**
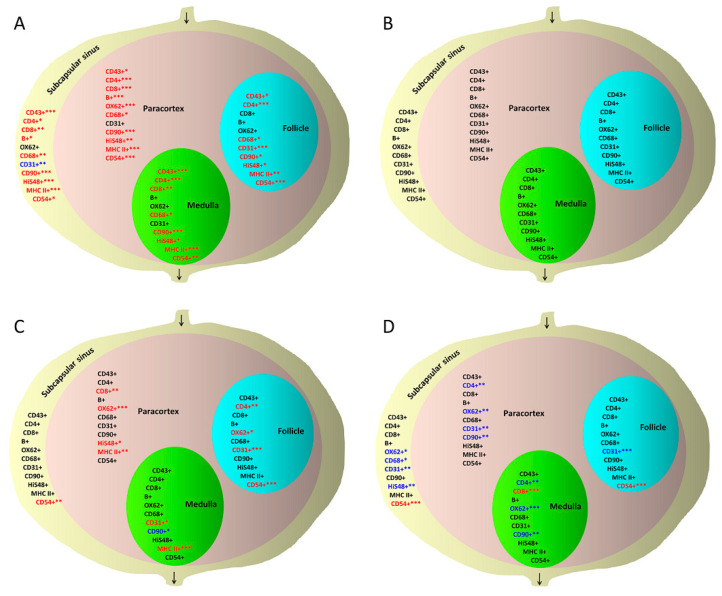
Quantitative changes in the surface of rat popliteal lymph node zones covered with immune cell subpopulations following *S. epidermidis* skin infection, assessed using the Microimage program (*n* = 8, 16 nodes). (**A**) Nodes after the initial massive skin infection with *S. epidermidis* with early evaluation (day 8) compared with controls administered 0.9% NaCl. (**B**) Nodes after the initial massive infection with late evaluation (day 28) compared with controls administered 0.9% NaCl. (**C**) Nodes after re-infection with *S. epidermidis* (evaluation on day 29) compared with the initial infection with late evaluation (day 28). (**D**) Nodes after re-infection compared with the initial infection with early evaluation (day 8). CD43+: T lymphocytes; CD4+: T helper lymphocytes, monocytes; CD8+: T cytotoxic lymphocytes; B+: B lymphocytes; OX62+: dendritic cells; CD68+: macrophages, monocytes; CD31+: PECAM-1, endothelial cells; CD90+: stem cells, thymocytes, immature B cells; HiS48+: granulocytes; MHC class II+: activated antigen-presenting cells; CD54+: dendritic, endothelial, ICAM-1+ cells. Red: increase in surface area of cells stained positive for the given antigen; blue: reduction in surface area of cells expressing the antigen of interest; black: no quantitative change. * *p* < 0.05; ** *p* < 0.01; *** *p* < 0.001.

In the medulla, after the initial massive skin infection with *S. epidermidis,* the surface area of T lymphocytes (CD43+), T helper lymphocytes (CD4+), T cytotoxic lymphocytes (CD8+), macrophages and monocytes (CD68+), granulocytes (HiS48+), stem cells, immature B cells and thymocytes (CD90+), activated antigen-presenting cells (MHC class II+), and CD54+ dendritic, endothelial, and ICAM-1+ cells (CD54+) significantly increased on day 8 compared with controls obtaining 0.9% NaCl. However, the differences were no longer observed on day 28. After secondary infection, a decrease in the area covered with T helper lymphocytes (CD4+), dendritic cells (OX62+), stem cells, thymocytes and immature B cells (CD90+), in addition to an increase in T cytotoxic lymphocyte (CD8+) area, were observed compared with the early effects (day 8) of the initial infection. However, when comparing the impact of secondary infection to late observations made on day 28 after the initial infection, other differences were observed: an increase in the area positive for endothelial cells (CD31+) and activated antigen-presenting cells (MHC class II+) and a decrease in the area covered by stem cells in addition to immature B cells and thymocytes (CD90+) (Supplementary Figure S11). The changes in the size of areas covered by cell subpopulations expressing key antigens in the rat lymph node zones upon massive, initial (day 8 and 28 of observation) and secondary (day 29) *S. epidermidis* infections are summarized in Figure 4.

## 4. Discussion

Knowledge regarding immune cells involved in eliminating skin saprophytes is incomplete. However, studying the effects of infection with skin saprophytic bacteria in the tissues located below the epidermis is challenging in humans due to the limited availability of the biological material. In our previous studies, some effects of several human skin saprophyte species on rat lymph nodes were assessed [25,29]. Notably, in rats, the most common human skin saprophyte, *S. epidermidis*, is also the normal colonizer [30]. Therefore, in the current study, we focused on *S. epidermidis* and significantly extended these observations.

The immune response depends on the ability of dendritic cells to recognize antigens in infected peripheral tissues, secrete inflammatory cytokines, capture and transport antigens to regional lymph nodes and present them to other immune cells [31]. The primary chemokine responsible for colonizing regional lymph nodes by activated dendritic cells via the afferent lymphatics is CCL21 [32]. PECAM-1, VCAM-1, ICAM-1, CD99, and L1CAM are other essential proteins involved in the migration of dendritic cells, antigen capture, interaction with macrophages, and the modulation of cytokine expression and changes in the vascular system [33]. The CCL19 and CCL21 chemokines present in HEVs are essential for targeting the naïve, memory, and regulatory subpopulations of CCR7+ T lymphocytes to the lymph nodes [34]. CCL19, secreted by activated dendritic cells in the nodes, enhances their interaction with T cells, enabling antigen presentation [35].

Indeed, when injected into the deep tissues of the limb, *S. epidermidis* induced activation of the immune system, and the response to re-infection was weaker than in the initial infection. After a 7-day course of bacteria injection that mimicked a 7-day massive infection, an increase was observed on day 8 in the total number of immune cells in the draining lymph nodes. However, these changes were no longer observed on day 28. One day after secondary infection, a re-increase in the number of immune cells, which was not as large as immediately after the massive initial infection, was observed. These results are consistent with previous observations [25].

Following the initial infection, one day after the bacteria administration cycle, flow cytometry analysis showed changes in some immune cell populations. There was an increase in the percentage of innate immune system cells such as CD68+ macrophages, ICAM-1-expressing cells, and CD54+ dendritic and endothelial cells, which is a normal response to bacterial infection. Conversely, the observed increase in the percentage of T cytotoxic CD8+ lymphocytes may indicate the activation of the effector cell response. Egawa et al. also showed that skin infection with *S. aureus* enhances the proliferation of cytotoxic T CD8+ lymphocytes due to the activation of dendritic cells residing in the draining lymph nodes [36]. Naik et al. showed that colonization of the skin by *S. epidermidis* stimulates IL-17A+CD8+ T cells populating the epidermis and strengthens innate barrier immunity. Commensal-specific T-cell responses result from the coordinated action of dendritic cells residing in the skin and are not associated with inflammation [12]. In addition, the reduction in the percentage of T helper CD4+ lymphocytes observed in the present study may result from the migration of these cells from lymphatic organs to the inflamed tissue. Pepper et al. showed that effector T helper 1 lymphocytes migrate from the central lymphoid organs after bacterial infection, which increases their chance of meeting the antigen. Some of these cells acquire features of the effector memory T helper 1 lymphocytes. In contrast, T follicular helper lymphocytes remain in the lymphoid organs as helper cells for B lymphocytes as long as the germinal centers in the follicles continue to respond to the antigen [37].

On day 28 of the experiment, after the initial infection with *S. epidermidis*, the cytometric evaluation revealed intensification of immune memory, as evidenced by an increase in the percentage of memory T helper lymphocytes (CD4+ CD45RC-) and effector memory T cytotoxic lymphocytes (CD8+ CD45RC- CD62L-). The presence of memory T helper CD4+ lymphocytes is essential for the memory B cell response and antibody production after repeated antigen encounters. By producing IFN-γ and IL-4, memory T helper lymphocytes activate macrophages. They also can silence secondary infection by releasing granzyme B or perforin and killing infected cells [38,39,40]. Furthermore, they enhance the functions of memory T cytotoxic CD8+ lymphocytes and participate in their formation [41]. Memory CD8+ T lymphocytes leave the lymph nodes and are transferred to the sites of infection, where they stay after infection resolution, protecting against subsequent infections. Upon antigen encounter, pathogen-specific tissue-resident memory T cells change the expression of genes encoding various antiviral and antibacterial factors, with IFN-γ being the most likely factor controlling these changes [42]. The reduction in the percentage of OX62+ dendritic cells on day 28 may indicate the involvement of these cells in antigen elimination because after antigen presentation to T cells, OX62+ dendritic cells were previously shown to undergo apoptosis, thus limiting the immune response [43]. Moreover, the lymph nodes draining sites with mature dendritic cells recruit CCR7- CD62L- effector T cells and CD8+ memory T cells. In the nodes, these cells interact with antigen-presenting dendritic cells and destroy them, further contributing to the termination of the primary immune response and limitation of the secondary response [44].

One day after re-infection with *S. epidermidis*, an increase in the percentage of CD8+ cytotoxic T cells was observed. Notably, CD43+ lymphocyte reaction was not detected after the initial infection; however, a significant increase in the percentage of this subpopulation was observed after secondary infection. Such an increase may indicate the activation of acquired cellular immunity. It is worth noting that although CD43 is present predominantly on T cells, it has also been identified in the B cell fraction. CD43+ B lymphocytes give rise to plasmablasts more efficiently than memory B cells [45,46]. Phenotyping of the human lymph node CD19+ B cell population showed increased CD27 protein expression during their differentiation in the germinal centers [47]. The binding of CD27 on B cells induces their differentiation into the memory lineage but inhibits terminal differentiation into plasma cells. Nevertheless, CD27 can be found on the surface of both memory B cells and plasma cells [47,48]. Indeed, after the secondary infection, we observed a significant increase in the percentage of CD19+ CD27+ cells representing the pool of memory B cells and plasmablasts. These cells were shown in mice to be involved in maintaining a long-term humoral response [48]. In response to subsequent challenges with the same antigen, central memory T lymphocytes residing in the secondary lymphoid organs rapidly proliferate and generate a large pool of effector T lymphocytes [49]. In lymphocyte B- and T-deficient Rag1^−/−^ mice with skin saprophytic flora present in the draining lymph nodes, after administration of lymphocytes isolated from healthy mice, Shen et al. showed a high proliferation of T CD8+, T CD4+, T γδ and IgM+ B lymphocytes. Many proliferating cells expressed CD44 and CD62L on their surface, which is the main feature of central memory T lymphocytes. Some cells expressed the CCR10 receptor, a marker of effector T lymphocytes determining their migration to the skin. In response to the skin saprophytic microorganisms, an intensive proliferation of CD8+ T cells compared with CD4+ T cells was demonstrated [11]. The high level of CD62L (L-selectin) on the surface of T cells allows them to enter secondary lymphoid organs. Using the rat model, Klinger et al. demonstrated a reduced expression of CD62L on CD4+ CD45RC- memory and CD4+ CD45RC+ resting T lymphocytes 24 h after reaching regional lymph nodes compared with peripheral blood T cells. Lowering the level of CD62L in the lymph node environment enables migration in various zones and from the lymph node to the site of inflammation. CD4+ CD45RC- CD62L- memory T cells can still be found in the circulation. However, the expression of other adhesion proteins LFA-1, ICAM-1, or integrin α4ß1 is increased in these cells, allowing them to enter the lymph nodes [50,51]. In accordance with these data, we showed that the percentage of effector memory T helper lymphocytes (CD4+ CD45RC- CD62L-) and cytotoxic memory T lymphocytes (CD8+ CD45RC-) was highest after the secondary *S. epidermidis* infection. The above observations and a reduction in the percentage of naïve B cells, CD54+, and MHC class II+ cells after the secondary infection indirectly indicate the effectiveness of memory cells to quickly neutralize the re-administered antigen.

Van der Aar et al. showed that epidermal Langerhans cells are characterized by a low capacity to internalize bacteria, process, and present bacterial antigens, in addition to the induction of antigen-specific T regulatory CD4+ Foxp3+ lymphocyte responses, thus contributing to the immune tolerance of saprophytes in this most superficial layer of the skin. In contrast, dermal dendritic cells efficiently recognize saprophytic bacteria and create an immune barrier in the skin against their deeper penetration, inducing a strong naïve and memory T CD4+ lymphocytes response [52]. Therefore, we hypothesize that in the present study, the lack of changes in the percentage of CD4+ CD25+ regulatory T cells in response to *S. epidermidis* infection was due to the short follow-up period.

Lymph nodes are organized structures topographically divided into functional compartments filled with cells responsible for antigen capture and transport (subcapsular sinus), antigen presentation (paracortex, follicles), cellular immunity (paracortex), humoral immunity (follicles, medulla), and antigen elimination (medulla) [53]. Subcapsular sinus macrophages constitute a barrier to penetrating pathogens by capturing them [54]. The macrophages present antigens to naïve B lymphocytes, CD8+ T lymphocytes, and natural killer T lymphocytes [55]. Lymph node follicles are the site of the development of germinal centers in response to an antigen. The germinal center microenvironment creates a niche in which activated B lymphocytes with follicular dendritic cells and T follicular helper lymphocytes participate in the generation of a long-lasting humoral immune response and are necessary for the production of high-affinity antibodies that protect against pathogens [56]. The paracortex of the lymph nodes is the site of T lymphocyte migration, antigen presentation, and generation of effector T cells [57]. Therefore, the morphometric evaluation of lymph node compartments for the presence of cells expressing antigens of interest allowed us to determine which cellular subpopulations in particular node zones are involved in eliminating *S. epidermidis*.

After the initial massive skin infection with *S. epidermidis*, a significant increase in the area occupied by cells expressing the majority of the studied antigens was observed in each functional zone of the node on day 8, indicating an intense stimulation of the immune system and a massive influx of cells of the innate immune system and naïve lymphocytes into the draining lymph node. An increase in the area covered by most of the studied cell subpopulations in the subcapsular sinus and follicles indicated an increase in the number of cells that capture and present antigens, helpers, and the *de novo* formation of memory and effector B lymphocyte clones producing specific antibodies. In the follicles, an increase in the area occupied by HiS48+ granulocytes was also observed. Neutrophils interact with B lymphocytes and plasma cells. After mobilization from the bone marrow, neutrophils reduce the early production of antigen-specific IgM and IgG antibodies in the lymph node by efficiently eliminating the antigen. In contrast, the decreased neutrophil number increases antibody production [58]. An increase in the area occupied by most of the cell types in the paracortex indicated a massive response of the naïve and effector T lymphocytes. Moreover, an increase in the area covered by cells expressing other cell type-specific antigens indicated stimulation of the influx of other immune cells from blood and lymph, including antigen-presenting cells and other innate immune cells. An increase in the surface area of HiS48+-positive cells was also observed in this node zone. Neutrophils shape the immune response by direct elimination of bacteria and presentation of the antigen to CD4+ T lymphocytes, by physical interaction with T lymphocytes, or by fusion with dendritic cells [58,59,60,61,62]. In the medulla, the increase in the surface area of cells expressing the majority of the studied antigens after primary antigen exposure was most likely due to the fact that lymphocyte output from lymph nodes is temporarily limited during inflammation, and different lymphocyte subtypes are restrained in the node [57]. This node zone also contains macrophages phagocytizing pathogens and neutrophils interacting with plasma cells upon antigen challenge [58]. Furthermore, in all lymph node zones, an increase in the area occupied by large cells expressing the CD90 antigen on their surface, including thymocytes, stem cells, and immature B cells was observed. These cells, selected in the thymus, migrate to secondary lymphoid organs, where they participate in the induction of immunity. CD90 is a protein that regulates adhesion, migration, proliferation, apoptosis, and cell communication and is involved in T cell activation, wound healing, and fibrosis [63].

On day 28 of the experiment, the morphometric evaluation showed that the size of the areas occupied by the main cell subpopulations in each functional lymph node zone returned to the initial state, indicating silencing of the immune response. Therefore, the initial stimulation with *S. epidermidis* triggered the innate and adaptive immunity involving all immune cell populations, which resulted in the effective elimination of the antigen.

After the secondary infection, the innate immune system cells and cytotoxic T CD8+ lymphocytes, helper CD4+ lymphocytes, and CD54+ cells quickly reacted. However, the surface area occupied by most of the studied cell subpopulations decreased or remained at the level observed on day 8 after the initial infection. These results are consistent with the results showing that the total cell count did not change in nodes after re-infection compared with observations on days 8 and 28. Assumedly, in the case of secondary infection, a high number of effector cells was not necessary because the elimination of the antigen was accomplished by antibodies produced by memory cells that were increased, as evidenced by flow cytometry.

The skin saprophytes are important because most microorganisms that live on human skin protect against pathogens [9,10]. As a result of microtrauma, the immune system is activated, and the skin and draining lymph nodes are likely colonized with antigen-specific immune cells [11]. Upon subsequent skin damage, cell clones of the adaptive immune response are activated, and infection, if not massive, proceeds without clinical symptoms. However, sub-epidermal administration of *S. epidermidis* saprophyte to rats, mimicking a 7-day massive infection, elicited a strong response in the lymph nodes from major cell subpopulations involved in generating an acquired, specific antigen response. The activation of many cell subpopulations in all lymph node compartments contributed to the efficient elimination of the antigen and formation of a pool of primary effector cells, including memory cells, based on observations made on day 28. After re-infection with *S. epidermidis*, the cellular response in the lymph nodes did not exceed the primary infection, and it was lower in the case of many cell subpopulations. The reduction in the secondary response may be due to the marked increase in the percentage of the immune memory B and T lymphocytes. However, this process was not influenced by regulatory T cells during the observation period. Therefore, the memory cells likely triggered a rapid humoral response, namely, the production of antibodies specific to the bacterial antigen recognized during the initial infection.

The present study has several limitations. Although the rats were bred under special sanitary standards (e.g., air conditioning with three types of filters, pass-through autoclaves, automatic washers for cages and bottles), previous contacts with saprophytes originating from their mothers cannot be excluded. Therefore, the initial 7-day infection with *S. epidermidis* could not be considered a primary infection. Instead, it was treated as a model of massive infection with the saprophytes, while repeated injection with the bacteria on day 28 of the experiment was a model of a secondary infection. Another limitation was that controls on days 28 and 29 were not included. However, the lymph nodes of controls injected with 0.9% NaCl and examined on day 8 did not differ from non-injected nodes (data not shown). Therefore, assumedly, if injections with 0.9% NaCl did not trigger an immune response on day 8 when the response was the strongest upon bacteria administration, the immune system would also not be activated later. This approach allowed us to limit the number of animals used in our experiments. Finally, we evaluated percentage changes but not changes in absolute numbers of cell subpopulations. Counting in the Bürker chamber showed an increase in the total immune cell number upon *S. epidermidis* infection. Therefore, in the flow cytometry analysis of 10,000 cells, the increase or decrease in the number of cells expressing the tested antigen can represent the actual change in cell number or a relative change caused by the infection-induced change in the number of other cells. We, therefore, decided that presenting percentage changes would be more appropriate. For the Microimage analysis, representative fields of view were selected for each node zone. However, as the size of some cell types can change upon infection, percentage changes in the surface covered by cells expressing a given antigen can only be used as a very rough surrogate for an increase or decrease in the number of these cells.

Conversely, the present study has several strengths. This is the first study demonstrating that after massive infection with *S. epidermidis*, most immune cell types present in the draining lymph nodes strongly respond by increasing their absolute numbers but without significant changes in proportions between cell types compared with control lymph nodes. In addition, for the first time, the percentage of memory B cells and plasmablasts CD19+ CD27+ were shown to increase in rats after re-infection with skin saprophytes, a process similar to this observed in other species [47,48,64,65].

## 5. Conclusions

In summary, skin saprophytic bacteria can become pathogenic when an imbalance exists between commensals and pathogens or when the epidermal barrier is severely damaged [66]. A thorough understanding of the natural *S. epidermidis* elimination by the lymph nodes can provide a basis for developing immunological therapies for individuals exposed to severe infections of deep tissues invaded by saprophytic bacterial flora, such as in cases of lymph stasis, acute dermato-lymphangio-adenitis, advanced atherosclerosis-related changes in lower extremities, chronic ulcers accompanying diabetes, and atopic dermatitis [23,67,68,69,70,71].

## Figures and Tables

**Table 1:** Determination of cell phenotypes.

Cell Type	Antigen (Rat Clone)
T lymphocytes	CD43+ (W3/13+)
T helper lymphocytes, monocytes	CD4+ (W3/25+)
T cytotoxic lymphocytes	CD8+ (OX8+)
B lymphocytes	B+
B lymphocytes	OX12+
B lymphocytes	CD45RA+ (OX33+)
Dendritic cells	OX62+
Macrophages, monocytes	CD68+ (ED1+)
PECAM-1, endothelial cells	CD31+
Stem cells, thymocytes, immature B lymphocytes	CD90+ (OX7+)
Granulocytes	HiS48+
Activated antigen-presenting cells	MHC class II+ (OX6+)
ICAM-1, dendritic cells, endothelial cells	CD54+
Activated T lymphocytes	CD43+ MHC class II+
Activated helper T lymphocytes	CD4+ MHC class II+
Activated cytotoxic T lymphocytes	CD8+ MHC class II+
T regulatory cells (Treg)	CD4+ CD25+
Memory B cells, plasmablasts	CD19+ CD27+
Memory T helper lymphocytes	CD4+ CD45RC-
Central memory T helper lymphocytes	CD4+ CD45RC- CD62L+
Effector memory T helper lymphocytes	CD4+ CD45RC- CD62L-
Memory T cytotoxic lymphocytes	CD8+ CD45RC-
Central memory T cytotoxic lymphocytes	CD8+ CD45RC- CD62L+
Effector memory T cytotoxic lymphocytes	CD8+ CD45RC- CD62L-

## Data Availability

Data are available on request from M.C.-J.

## Supplementary Materials

The following supporting information can be downloaded at: https://www.mdpi.com/article/10.3390/biomedicines10051021/s1, Supplementary Table S1: Antibodies used for immunocytochemistry and immunohistology; Supplementary Figure S1: Afferent lymphatics and subcapsular sinus of the rat popliteal lymph node in initial massive *S. epidermidis* infection (days 1–7) with early node’s evaluation (day 8); Supplementary Figure S2: Subcapsular sinus of the rat popliteal lymph node stained for the presence of migrating dendritic cells (OX62+, red); Supplementary Figure S3: Subcapsular sinus of the rat popliteal lymph node stained for the presence of macrophages and monocytes (CD68+, red); Supplementary Figure S4: Subcapsular sinus of the rat popliteal lymph node stained for the presence of granulocytes (HiS48+, red); Supplementary Figure S5: The rat popliteal lymph node with the visible follicle stained for the presence of T helper lymphocytes and monocytes (CD4+, red); Supplementary Figure S6: The rat popliteal lymph node with the visible follicle stained for the presence of activated antigen-presenting cells (MHC class II+, red); Supplementary Figure S7: Paracortex of the rat popliteal lymph node stained for the presence of T lymphocytes (CD43+, red); Supplementary Figure S8: Paracortex of the rat popliteal lymph node stained for the presence of T cytotoxic lymphocytes (CD8+, red); Supplementary Figure S9: Paracortex of the rat popliteal lymph node stained for the presence of activated antigen-presenting cells (MHC class II+, red); Supplementary Figure S10: Paracortex of the rat popliteal lymph node in initial massive *S. epidermidis* infection (days 1–7) with early node’s evaluation (day 8) stained for the presence of large stem cells, thymocytes and immature B cells (CD90+, red); Supplementary Figure S11: The medulla of the rat popliteal lymph node in initial massive *S. epidermidis* infection (days 1–7) with early node’s evaluation (day 8).
