# Supplementary material for: Towards Understanding the Lymph Node Response to Skin Infection with Saprophytic Staphylococcus epidermidis
Source: Biomedicines. 2022 Apr 28;10(5):1021. doi: 10.3390/biomedicines10051021 (PMC9138836; doi:10.3390/biomedicines10051021)
Supplement: Supplementary file 1 [file biomedicines-10-01021-s001.zip › biomedicines-1624197-SM.pdf]

Table S1. Antibodies used for immunocytochemistry and immunohistology.

CD (antigen)	Rat, clone	Manufacturer	Concentration	Dilution
CD43	W3/13	S	1.0 mg/ml	1:100
CD4	W3/25	S	1.0 mg/ml	1:100
CD4 FITC	W3/25	S	0.1 mg/ml	1:100
CD4 PECy-5	OX35	BD	0.2 mg/ml	1:100
CD8	OX8	S	1.0 mg/ml	1:100
CD8a PerCP	OX8	BD	0.1 mg/ml	1:100
B	RLN-9D3	S	1.0 mg/ml	1:100
Ig k-chain	OX12	S	1.0 mg/ml	1:100
CD45RA	OX33	S	1.0 mg/ml	1:100
CD25 PE	OX39	BD	0.2 mg/ml	1:100
-	OX62	S	0.1 mg/ml	1:30
CD68	ED1	S	0.1 mg/ml	1:70-1:100
CD68 PE	ED1	S	0.1 mg/ml	1:50
CD31 (PECAM-1)	TLD-3A12	S	1.0 mg/ml	1:50
-	HiS48	S	na	1:100
CD90	OX7	S	1.0 mg/ml	1:100
MHC CLASS II RT1B	OX6	S	1.0 mg/ml	1:100
MHC CLASS II RT1B	OX6 FITC	S	1.0 mg/ml	1:100
CD54 (ICAM-1)	1A29	S	1.0 mg/ml	1:50
CD19 PE	R-20	SCB	1.0 mg/ml	1:100
CD27	LG.3A10	BD	0.1 mg/ml	1:100
CD45RC PE	OX22	BD	0.2 mg/ml	1:100
CD62L FITC	OX85	S	1.0 mg/ml	1:100
IgG1	-	S	0.1 mg/ml	1:10
IgG2a	-	S	0.1 mg/ml	1:10
IgG1 PE	R3-34	BD	0.1 mg/ml	1:10
IgG1 FITC	R3-34	BD	0.1 mg/ml	1:10
IgG1 PerCP	RTK2071	BL	0.2 mg/ml	1:10
IgG2a PE Cy-5	R35-95	BD	0.2 mg/ml	1:10
IgG PE	Poly24030	BL	0.1 mg/ml	1:10
IgG FITC	HTK888	BL	0.5 mg/ml	1:10

S: Serotec Ltd, Kidlington, United Kingdom; BD: Becton Dickinson Biosciences Pharmingen, San Jose, CA, USA; SCB: Santa Cruz Biotechnology, Dallas, TX, USA; BL: Bio Legend, San Diego, CA, USA.

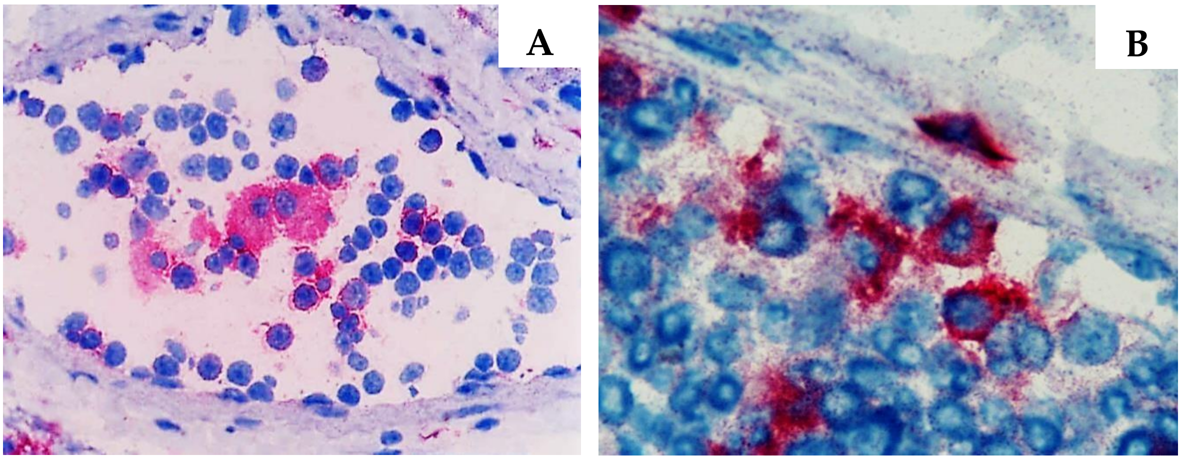

Figure S1. Afferent lymphatics and subcapsular sinus of the rat popliteal lymph node in initial massive *S. epidermidis* infection (days 1-7) with early node's evaluation (day 8). A: T helper lymphocytes and monocytes (CD4+, red) entering the popliteal lymph node via afferent lymphatics, magnification 600 x. B: Migrating dendritic cells (OX62+, red) in the subcapsular sinus, magnification 1000 x.

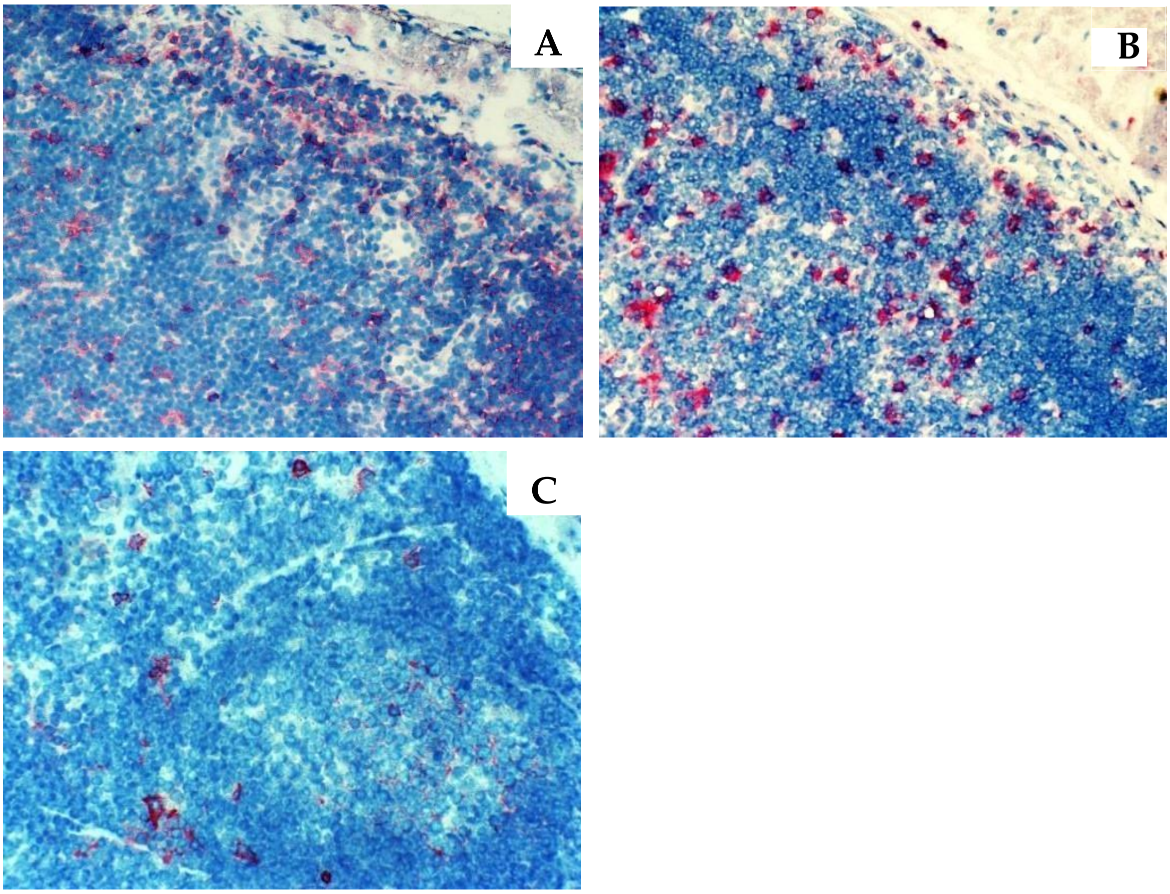

Figure S2. Subcapsular sinus of the rat popliteal lymph node stained for the presence of migrating dendritic cells (OX62+, red). A: The node of the control animal receiving 0.9% NaCl (days 1-7, node evaluation on day 8). B: The node after initial massive *S. epidermidis* infection (days 1-7) with early node evaluation (day 8). C: The node after secondary *S. epidermidis* infection (day 28, node evaluation on day 29). Magnification 200 x.

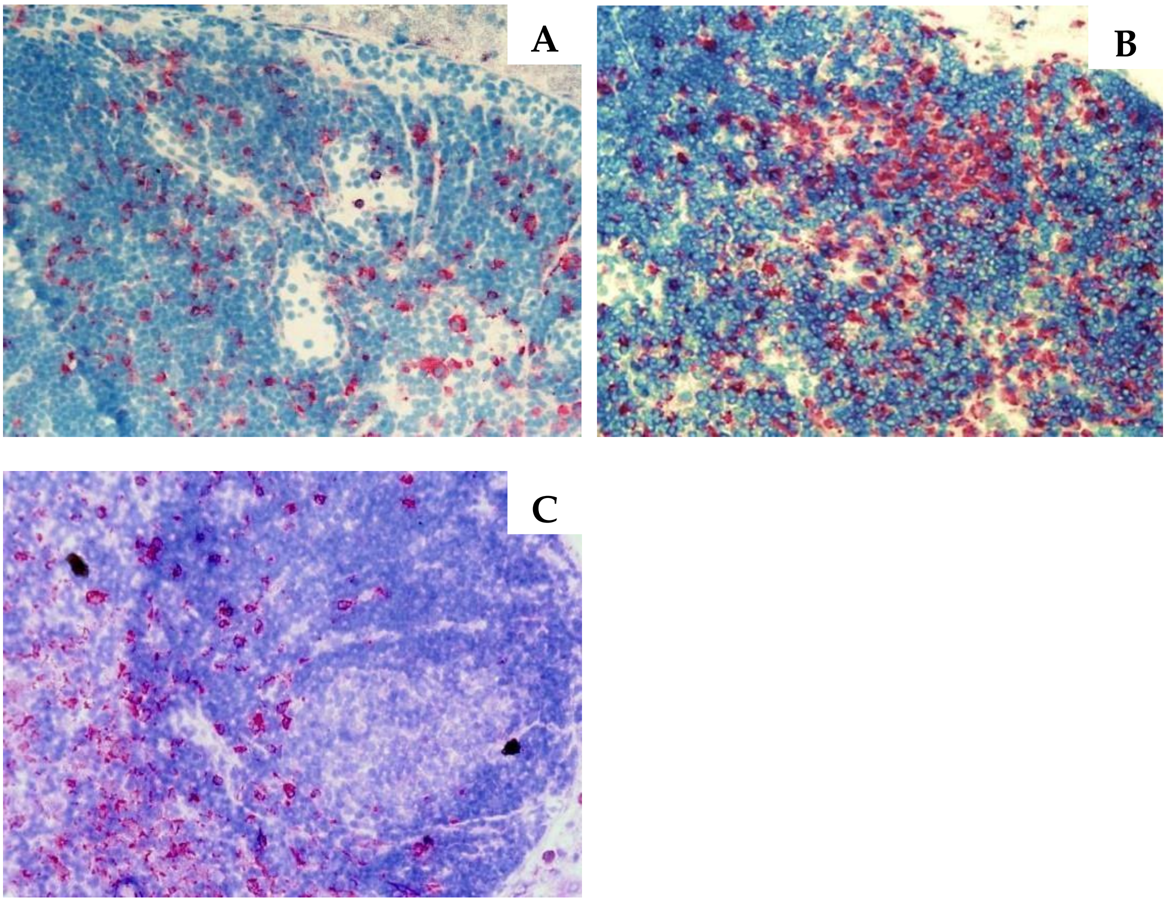

Figure S3. Subcapsular sinus of the rat popliteal lymph node stained for the presence of macrophages and monocytes (CD68+, red). A: The node of the control animal receiving 0.9% NaCl (days 1-7, node evaluation on day 8). B: The node after initial massive *S. epidermidis* infection (days 1-7) with early node evaluation (day 8). C: The node after secondary *S. epidermidis* infection (day 28, node evaluation on day 29). Magnification 200 x.

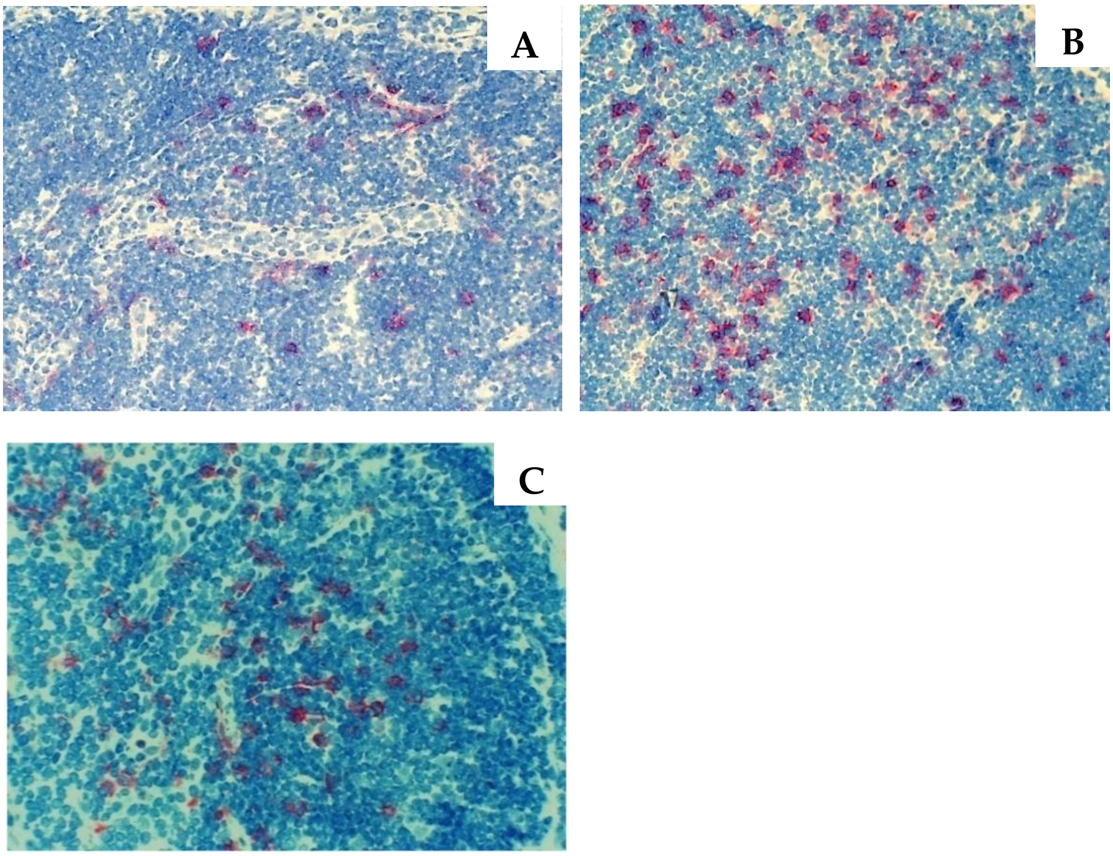

Figure S4. Subcapsular sinus of the rat popliteal lymph node stained for the presence of granulocytes (HiS48+, red). A: The node of the control animal receiving 0.9% NaCl (days 1-7, node evaluation on day 8). B: The node after initial massive *S. epidermidis* infection (days 1-7) with early node evaluation (day 8). C: The node after secondary *S. epidermidis* infection (day 28, node evaluation on day 29). Magnification 200 x.

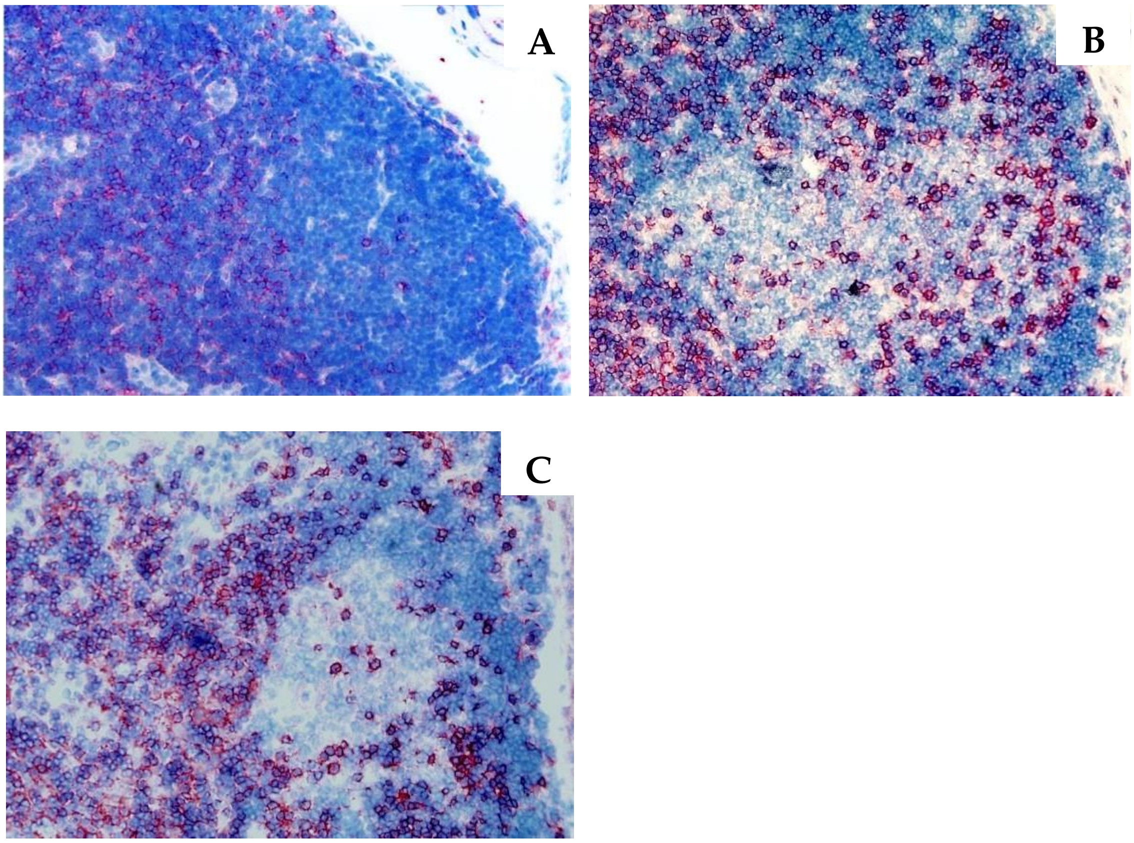

Figure S5. The rat popliteal lymph node with the visible follicle stained for the presence of T helper lymphocytes and monocytes (CD4+, red). A: The node of the control animal receiving 0.9% NaCl (days 1-7, node evaluation on day 8). B: The node after initial massive *S. epidermidis* infection (days 1-7) with early node evaluation (day 8). C: The node after secondary *S. epidermidis* infection (day 28, node evaluation on day 29). Magnification 200 x.

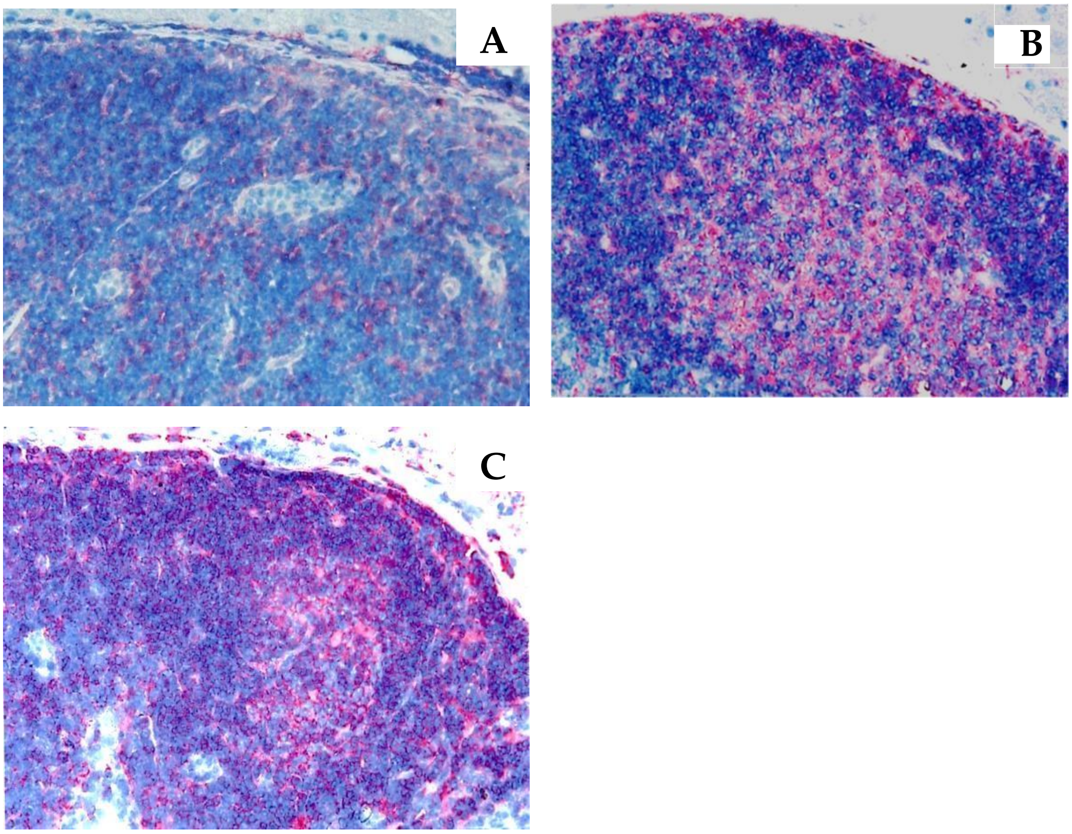

Figure S6. The rat popliteal lymph node with the visible follicle stained for the presence of activated antigen-presenting cells (MHC class II+, red). A: The node of the control animal receiving 0.9% NaCl (days 1-7, node evaluation on day 8). B: The node after initial massive *S. epidermidis* infection (days 1-7) with early node evaluation (day 8). C: The node after secondary *S. epidermidis* infection (day 28, node evaluation on day 29). Magnification 200 x.

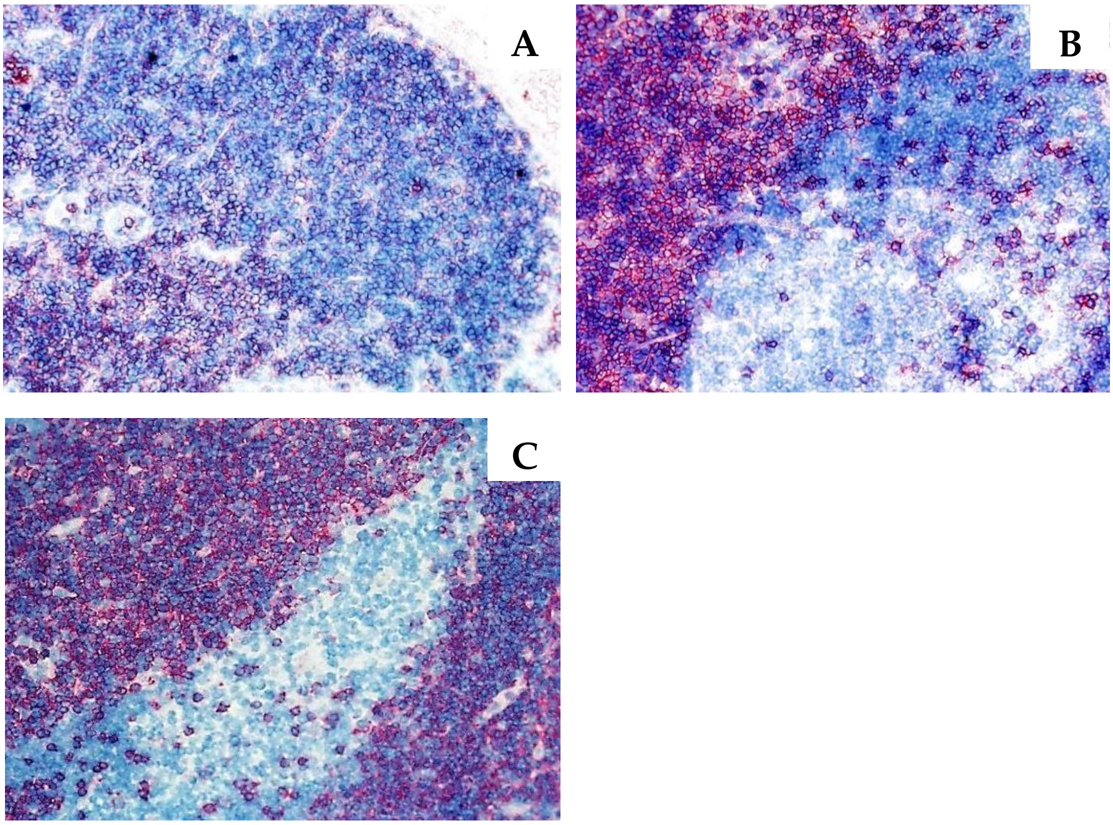

Figure S7. Paracortex of the rat popliteal lymph node stained for the presence of T lymphocytes (CD43+, red). A: The node of the control animal receiving 0.9% NaCl (days 1-7, node evaluation on day 8). B: The node after initial massive *S. epidermidis* infection (days 1-7) with early node evaluation (day 8). C: The node after secondary *S. epidermidis* infection (day 28, node evaluation on day 29). Magnification 200 x.

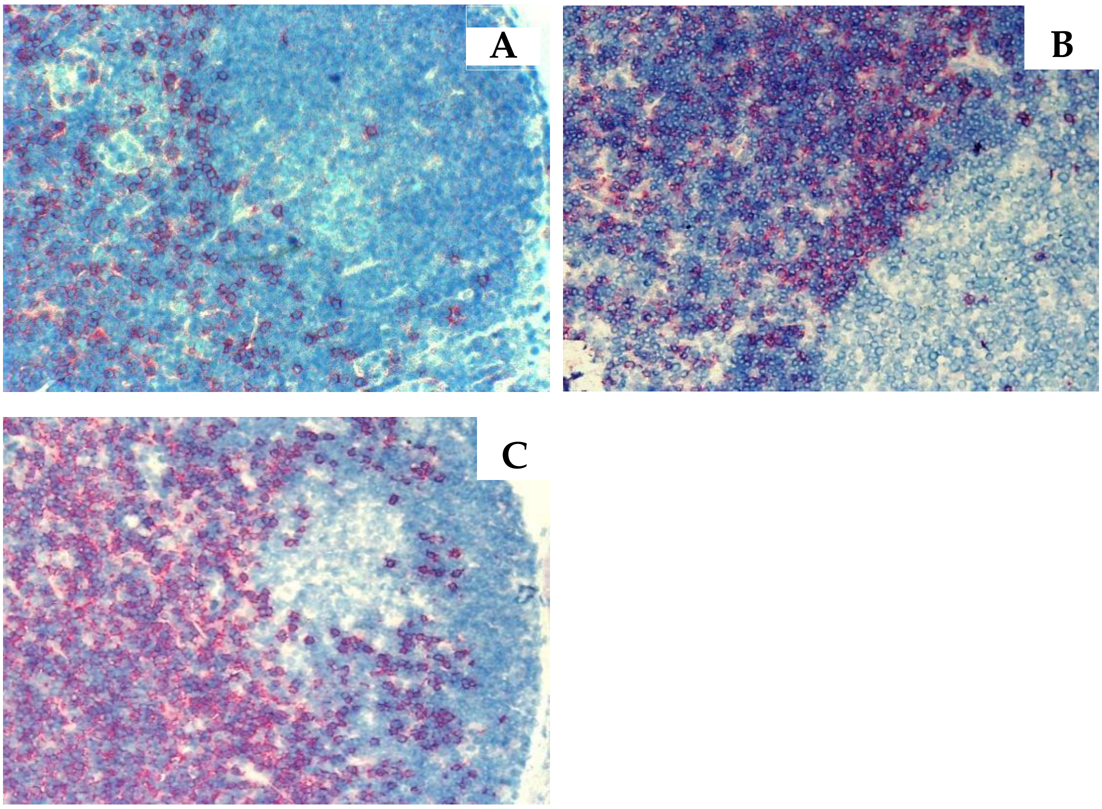

Figure S8. Paracortex of the rat popliteal lymph node stained for the presence of T cytotoxic lymphocytes (CD8+, red). A: The node of the control animal receiving 0.9% NaCl (days 1-7, node evaluation on day 8). B: The node after initial massive *S. epidermidis* infection (days 1-7) with early node evaluation (day 8). C: The node after secondary *S. epidermidis* infection (day 28, node evaluation on day 29). Magnification 200 x.

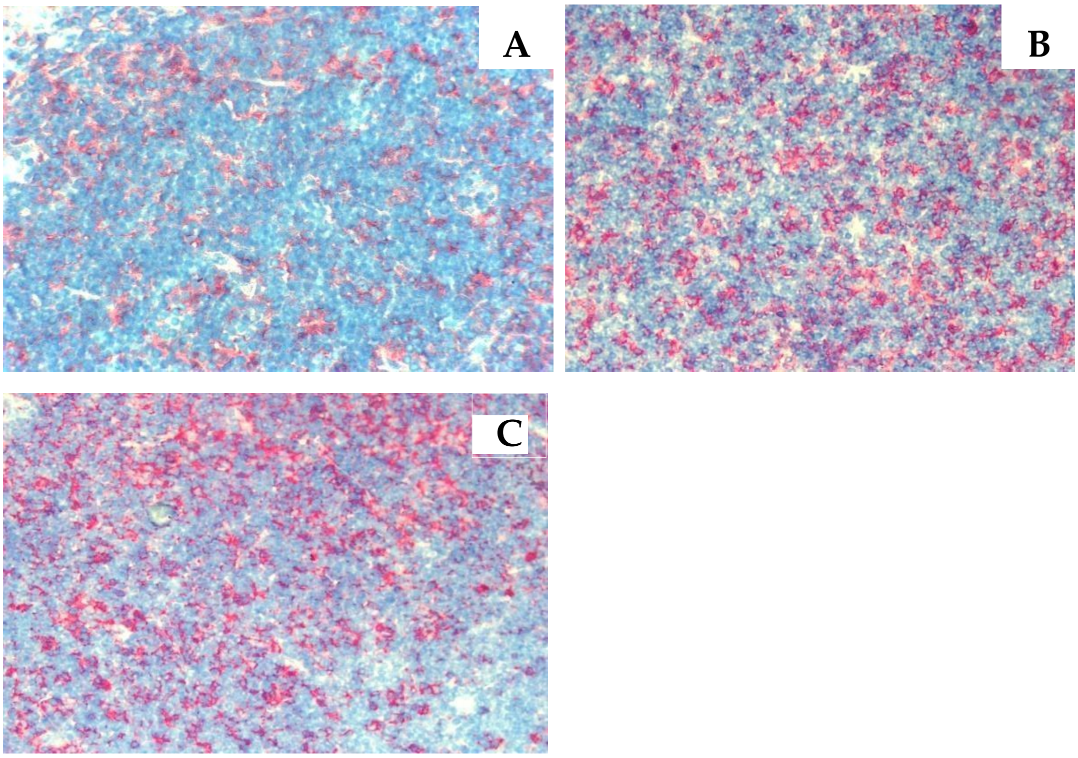

Figure S9. Paracortex of the rat popliteal lymph node stained for the presence of activated antigen-presenting cells (MHC class II+, red). A: The node of the control animal receiving 0.9% NaCl (days 1-7, node evaluation on day 8). B: The node after initial massive *S. epidermidis* infection (days 1-7) with early node evaluation (day 8). C: The node after secondary *S. epidermidis* infection (day 28, node evaluation on day 29). Magnification 200 x.

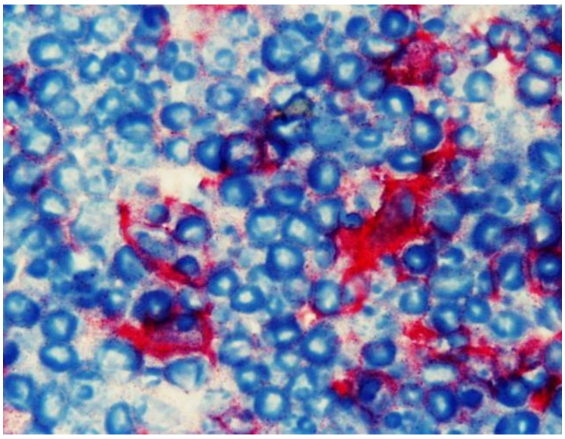

Figure S10. Paracortex of the rat popliteal lymph node in initial massive *S. epidermidis* infection (days 1-7) with early node's evaluation (day 8) stained for the presence of large stem cells, thymocytes and immature B cells (CD90+, red). Magnification 1000 x.

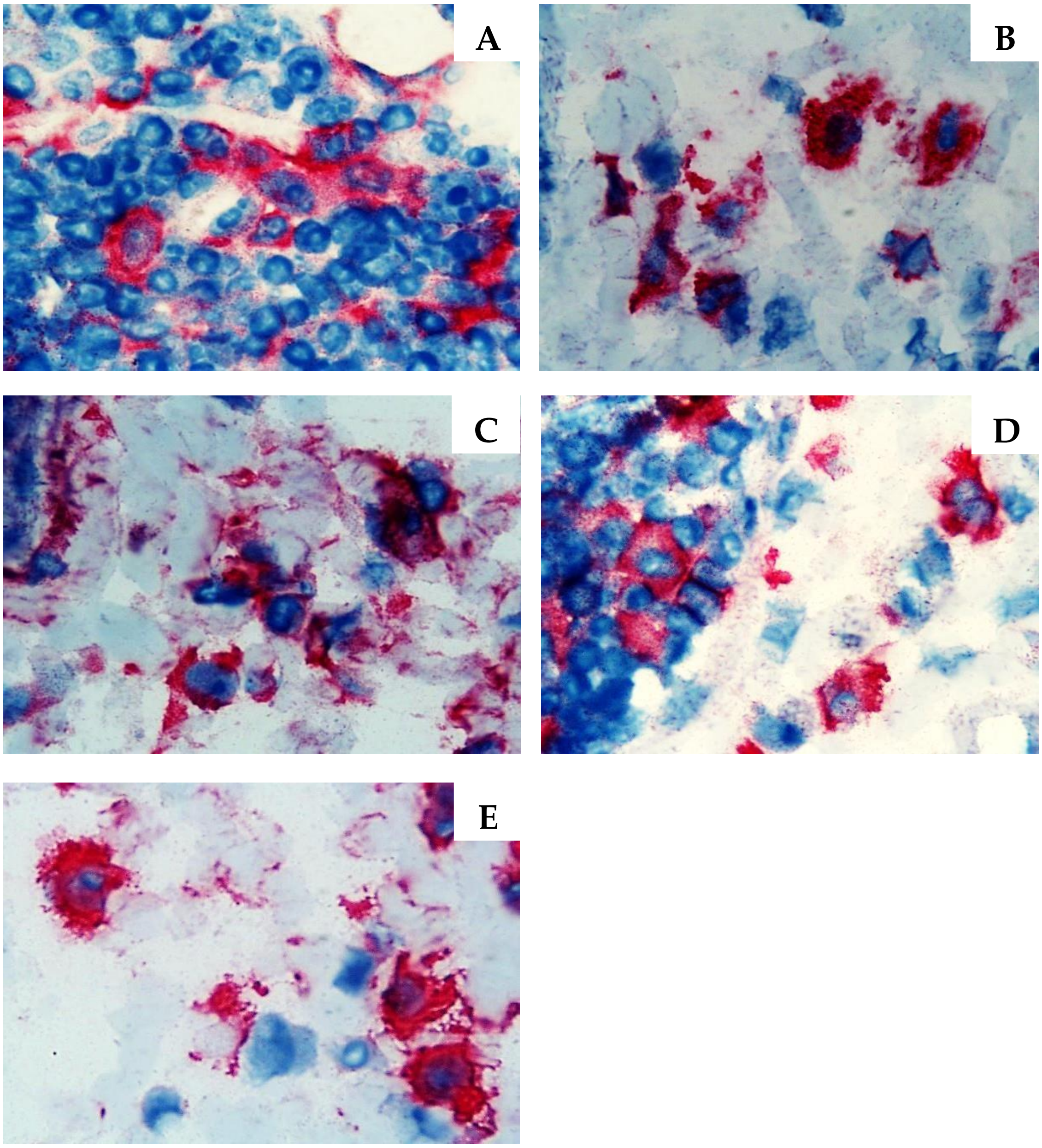

Figure S11. The medulla of the rat popliteal lymph node in initial massive *S. epidermidis* infection (days 1-7) with early node's evaluation (day 8). The node stained for: A: Dendritic cells (OX62+, red). B: Macrophages and monocytes (CD68+, red). C: Stem cells, thymocytes and immature B cells (CD90+, red). D: T helper lymphocytes and monocytes (CD4+, red). E: Activated antigen-presenting cells (MHC class II+, red). Magnification 1000 x.
